# Association of Platelet-Rich Plasma and Auto-Crosslinked Hyaluronic Acid Microparticles: Approach for Orthopedic Application

**DOI:** 10.3390/polym11101568

**Published:** 2019-09-26

**Authors:** Andréa Arruda Martins Shimojo, Adriana da Silva Santos Duarte, José Fábio Santos Duarte Lana, Ângela Cristina Malheiros Luzo, Ana Rita Fernandes, Elena Sanchez-Lopez, Eliana Barbosa Souto, Maria Helena Andrade Santana

**Affiliations:** 1 Department of Engineering of Materials and Bioprocesses, School of Chemical Engineering, University of Campinas, Campinas 13083-852, SP, Brazil; 2 Department of Pharmaceutical Technology, Faculty of Pharmacy, University of Coimbra (FFUC), Pólo das Ciências da Saúde, Coimbra 3000-548, Portugal; 3 Haematology and Hemotherapy Center, Umbilical Cord Blood Bank, University of Campinas, Campinas 13083-878, SP, Brazil; 4 Institute of Bone and Cartilage (IOC), Indaiatuba 13334-170, SP, Brazil; 5 Department of Pharmacy, Pharmaceutical Technology and Physical Chemistry. Faculty of pharmacy and Food Sciences. University of Barcelona. Institute of Nanoscience and Nanotechnology (IN2UB), 08028 Barcelona, Spain; 6 Centro de Investigación biomédica en red de enfermedades neurodegenerativas (CIBERNED), 08028 Barcelona, Spain; 7 CEB–Centre of Biological Engineering, University of Minho, Campus de Gualtar, 4710-057 Braga, Portugal

**Keywords:** platelet-rich plasma, polyelectrolyte complexes, auto-crosslinked hyaluronic acid, chitosan, cell proliferation

## Abstract

Platelet-rich plasma (PRP) associated with high molecular weight hyaluronic acid (HA) has been clinically used for tissue regeneration in orthopedics. Despite the recognized beneficial clinical outcomes (e.g., early pain control, improvement of patients’ functional limitation and longer-term effectiveness compared to PRP and HA alone in mild and moderate osteoarthritis treatments), its use is still challenging and controversial due to lack of standardization of association practical protocols. Moreover, most studies neglect the matrix structure, that generates the ultimate properties of the association among platelets, fibrin network and the microparticles. In the present work, we aimed to analyze the influence of the PRP/HA association with a controlled matrix structure on the stability, rheological behavior, release of growth factors and in vitro proliferation of human adipose-derived mesenchymal cells (h-AdMSCs). The attenuation of the negative charge of HA was also evaluated. Pure PRP (P-PRP) (i.e., plasma enriched with platelets and poor in leukocytes) was prepared by centrifugation and activated with serum and calcium chloride (_A_P-PRP). Autocrosslinked hyaluronic acid (AHA) was prepared by organocatalyzed auto-esterification and structured in microparticles (_MP_AHA) by shearing. The attenuation of the negative charge of _MP_AHA was performed with chitosan (CHT) by polyelectrolyte complexation yielding _MP_AHA-CHT. The results showed that microparticles (MPs) have viscoelastic properties, extrusion force and swelling ratio appropriate for injectable applications. The association of _A_P-PRP with the controlled structure of _MP_AHA and _MP_AHA-CHT formed a matrix composed of platelets and of a fibrin network with fibers around 160 nm located preferably on the surface of the MPs with an average diameter of 250 μm. Moreover, _A_P-PRP/_MP_AHA and _A_P-PRP/_MP_AHA-CHT associations were non-toxic and supported controlled growth factor (PDGF-AB and TGF-β1) release and in vitro proliferation of h-AdMSC with a similar pattern to that of _A_P-PRP alone. The best h-AdMSC proliferation was obtained with the _A_P-PRP/_MP_AHA-CHT_75:25_ indicating that the charge attenuation improved the cell proliferation. Thus, the association of _A_P-PRP with the controlled structure of HA can be a valuable approach for orthopedic applications.

## 1. Introduction

Platelet-rich plasma (PRP) is a concentrate of platelets and other components of plasma, which has been widely used in regenerative medicine [1,2,3,4]. The regenerative potential of PRP is based on the release of growth factors that occurs with platelet degranulation. The activation of PRP with autologous serum and CaCl_2_ promotes the release of growth factors, which stimulate cell proliferation and differentiation.

More recently, PRP has been associated with high molecular weight hyaluronic acid (HA), mainly for orthopedic therapies [2,5]. HA is a glycosaminoglycan formed by a variable number of repeating units of D-glucuronic acid and N-acetylglucosamine. Besides its role in lubrication and joints protection against shocks, HA exhibits anti-inflammatory effects, relieves pain, provides signalization of specific receptors (such as CD44), activates intrinsic repair processes of the cartilage, and normalizes the endogenous production of HA [2,5,6,7].

Although several clinical reports have shown that the association of PRP and HA promotes tissue regeneration, improves the mobility of patients, relieves pain and reduces the risk of infection in orthopedics treatment, studies demonstrating its effectiveness are still limited and controversial [8,9]. Most studies report the use of growth factors and cytokines from PRP only, without considering the fibrin network and its interactions with biomaterials as scaffolds. An ideal scaffold must provide a frame for the cell attachment, proliferation and differentiation. The biomaterials should be non-toxic and stable, having a pore size and/or porosity large enough to ensure cellular nutrition but not too large to prevent cell migration. Furthermore, the surface topography and chemistry (wettability, softness, stiffness, and roughness), microstructure (porosity, pore size, pore shape, and interconnectivity) and mechanical properties have a significant influence on the processes involved in tissue regeneration [10].

Moreover, the standardization and optimization of this association (i.e., mass ratio PRP/HA), molar mass of HA, need for crosslinking of HA, the structure of HA (hydrogel, micro or nanoparticles, fibers), and protocols of mixture of PRP and HA, are still a challenge. Despite the numerous studies described in the literature, as far as we know, there is only one study associating PRP/HA-based on a matrix structure (Supplementary Figure S1) formed by platelets, fibrin network and/or uses crosslinked HA microparticles [11]. 

Thus, this study, which also considers the structure of the matrix (platelets + fibrin network + microparticles), is the starting point to better understand the complex processes involved in this association and in tissue healing. The crosslinking of HA prevents the rapid degradation of HA chains by enzymatic action and/or by reactive oxidative species (ROS), and to extends the action over time. Moreover, the crosslinking improves the mechanical properties, such as dumping and viscoelasticity of HA, when compared to the non-crosslinked one. 

The structuring of microparticles enables the increase of the surface area for cell spreading and adhesion, contributing to the proliferation and control of matrix structure. Moreover, the reduction of the negative charge by the addition of chitosan, improves cell adhesion on the microparticles, while the fibrin network from activated PRP acts as the natural scaffold favoring the cell adhesion and proliferation, and improving the mechanical properties and the stability of matrix (HA microparticles + fibrin network).

However, Shimojo et al. used 1,4-butanediol diglycidyl ether (BDDE) as crosslinkers [11]. Although the association _A_P-PRP/HA_BDDE_ structured in microparticles has benefited the physicochemical and mechanical properties compared to _F_HA, the release of growth factors was similar to fibrin network from _A_P-PRP and the mesenchymal cells proliferation (which are progenitors for bone and cartilage mature cells contributing to bone and cartilage repair). 

Our hypothesis is that a controlled matrix structure will increase the stability of association P-PRP/HA preventing phase separation of free HA and will enable standardization of protocols for clinical applications. Thus, the present work aimed to analyze the influence of the P-PRP/HA association with controlled matrix structure on the stability, rheological behavior, delivery of growth factors and in vitro proliferation of human-adipose mesenchymal cells (h-AdMSCs). The control of matrix structure was obtained by association of fibrin network from activated P-PRP (_A_P-PRP) with auto-crosslinked HA microparticles (_MP_AHA). Ren et al. [12] observed that HA may reduce cell adhesion due to the negative charge of molecular chain, while Maroudas [13] demonstrated that it is not the negative charge that impairs cell adhesion but its density. Thus, the effects of attenuation of the negative charged HA by surfacing it with chitosan were also evaluated using the mass ratios AHA/CHT 100/0 (very negative), 90/10 (neutral), 75/25 (positive), and 50/50 (very positive) [12,13]. The main advantage of auto-crosslinking, obtained by organocatalyzed auto-esterification, is to provide stability to the HA matrix in the absence of chemical crosslinkers and to be used for different types of micro/nanoparticles [14,15,16,17,18,19,20,21,22,23].

## 2. Materials and Methods

### 2.1. Materials

Hyaluronic acid (HA) in sodium salt form (*M*_w_ = 2 × 10^5^ Da) was obtained from Spec-Chem Ind. (Nanjing, China). Chitosan (*M*_w_ = 4 × 10^5^ Da, degree of acetylation = 17.0 ± 0.7%) was purchased from Polymar^®^ (Fortaleza, CE, Brazil) and purified according to a protocol described by Nasti et al. [24]. Other chemicals were of reagent grade and were used without any further purification. 

### 2.2. Methods

#### 2.2.1. Preparation and Characterization of Auto-Crosslinked Hyaluronic Acid (AHA)

Initially, in order to prepare AHA, the intermediary tetrabutylammonium hyaluronate (HA-TBA) was prepared. In brief, an aqueous solution of 0.5% (g/100 mL) sodium hyaluronate (Na-HA, pH 5.86) was stirred in strongly acidic ion exchange resin Dowex^®^ 50-8WX-400 mesh (Sigma-Aldrich, St. Louis, MO, USA) for 8 h to form hyaluronic acid (H^+^-HA, pH ~3.80). The suspension was centrifuged in a Rotina 380R centrifuge (Hettich^®^ Zentrifugen, Tuttlingen, Germany) at 10,000 rpm for 10 min to remove the resin, and it was then neutralized with tetrabutylammonium hydroxide (TBA-OH) 0.2 mol/L (Vetec^®^, Duque de Caxias, Rio de Janeiro, Brazil) to form the quaternary ammonium salt of HA (TBA-HA, pH 6.96). The solution was then frozen and lyophilized in a Liobras L101 (Liobras, São Carlos, SP, Brazil) for 48 h. 

AHA was prepared by esterification reaction organocatalyzed with trimethylamine (Supplementary Figure S2) according to the protocol described by Bellini et al. [25] and Schanté et al. [26]. Briefly, HA-TBA (10 mEq of monomeric units) was solubilized in 50 mL of dimethyl sulfoxide (DMSO) at 25 °C. Triethylamine (0.5 mEq) was added, and the resulting solution was stirred for 30 min. A solution of 2-chloro-1-methyl pyridinium iodide (CMPI) (0.5 mEq) (Sigma-Aldrich, St. Louis, MO, USA) in 15 mL of DMSO was slowly added dropwise over 1 h, and the mixture was kept at 30 °C for 15 h. A solution of sodium chloride 2.5% (g/100 mL) was then added, and the resulting mixture was poured slowly into 150 mL of acetone, maintaining continuous stirring. The obtained precipitate was centrifuged at 10,000 rpm for 10 min, washed thrice in 100 mL of 5:1 acetone: water and then thrice with 100 mL of acetone, and vacuum-dried for 24 h at 30°C. 

Chemical characterization of AHA was performed by Fourier transform infrared (FTIR). Measurements were performed in a Thermo Scientific Nicolet model 6700 (Thermo Scientific Nicolet™, Waltham, MA, USA), in the ATR mode with accessory SMART OMNI-SAMPLER, in the spectral range of 4000–675 cm^−1^ with a resolution of 4 cm^−1^ over 64 scans. 

#### 2.2.2. Preparation and Characterization of Microparticles of AHA (_MP_AHA)

_MP_AHA microparticles were obtained by high shear homogenization in an Ultra-Turrax T25 homogenizer (IKA Labortechnik, Staufen, Germany) at 18,000 rpm for 20 min of plane hydrogel swelling in ultrapure water. _MP_AHA were characterized by effective crosslink density Flory-Rehner calculations, average diameter, degradation time, rheology, extrusion force, swelling ratio, and cytotoxicity.

*Effective crosslink density Flory-Rehner calculations.* Effective crosslink density was evaluated by measuring the volumetric swelling and using a simplified version of the Flory and Rehner equation. The value used for chitosan-water interaction parameter (χ) was 0.439 [27].

*Degradation time.* The gravimetric method described by Tang *et al.* [28] was used to estimate the degradation time of the MPs through measurements of the remaining weight. The assay was carried out with the MPs in phosphate buffer saline solution (PBS, pH 7.2) (LB Laborclin, Pinhais, PR, Brazil) at 37 °C.

*Average diameter.* The average diameter of MPs was measured in water. The measurements were performed with Malvern Mastersizer S laser diffraction (Malvern Instruments Ltd, Malvern, UK). The standard deviation was calculated from ten consecutive measurements.

*Rheology.* Rheological measurements were performed in the oscillatory and steady regime at 25 °C using a parallel plate geometry of 20 mm. Oscillatory measurements were conducted in the linear region with a stress of 1.188 Pa and in the frequency range of 0.1 to 10 Hz. Steady shear measurements were carried out at shear rates of 0.1–50 s^−1^. All rheological measurements were performed on a rheometer Haake RheoStress 1 (Haake, Karlsruhe, Germany). The parameters were calculated from the rheological curves, such as complex viscosity (G*), tan δ and power-law index (n) (Equations 1, 2, and 3, respectively).

(1) $$G^{*}=((G^{\prime }{)}^{2}\;+\;(G^{\prime \prime }{)}^{2}{)}^{1/2}$$

(2) $$\tan\delta =\frac{G^{\prime \prime }}{G^{\prime }}$$

(3) $$\left(ƞ\;=K\;\gamma^{n-1}\right)$$

*Extrusion force.* The MPs were firstly loaded in 1-mL plastic syringes with 30-gauge needles. Afterward, the force required to extrude was measured in an MTS 810 Servo-hydraulic Universal Testing Machine (MTS Systems Corporation, Eden Prairie, MN, USA) (Load Cell 1.5 kN) at 25 °C at a 5.0 mm/min extrusion rate.

*Swelling ratio.* Swelling measurements of the MPs were performed in PBS at 37°C for 72 h, according to Shu *et al.* [29]. The swelling ratio (SR) was calculated using the following Equation 4.

(4) $$SR=\frac{Ws}{Wd}$$

where ws and wd are the weights of the scaffolds in the swelled state and the dry state, respectively.

*Cytotoxicity.* The cytotoxicity of the MPs expressed as the viability of h-AdMSCs cells was carried out by exposing them to cell culture at 37 °C for 24 h (ISO 10993-5: "Tests for Cytotoxicity—In Vitro Methods"). Then, cell viability was evaluated using the MTT assay (3-[4,5-dimethyl-thiazol-2-yl]-2,5-diphenyltetrazolium bromide) (MTT, Sigma-Aldrich, St. Louis, MO, USA), according to the modified Mosmann method [30]. 

#### 2.2.3. Attenuation of Negative Charge of AHA

The attenuation of negative charges of AHA was performed by chitosan (CHT) addition. Polyelectrolyte complexes of AHA-CHT (Supplementary Figure S3) were produced in pH 4.0 by complex coacervation between amino groups (NH_3_^+^) of CHT (p*Kb* ~ 6.5) and carboxylic groups (COO^−^) of AHA (p*Ka* ~ 2.9) [31]. Briefly, AHA powder was swollen with a solution of CHT diluted acetic acid 1% (g/100 mL) using a final polymer concentration of 1% (*w*/*w*). The reaction mixture at room temperature was kept under stirring at 1,000 rpm overnight. Then, the formed precipitates were washed with water and phosphate buffer saline (PBS) to pH 7 (pH 6.6–6.8). The mass ratio AHA:CHT used in the microparticles were 90:10 (AHA-CHT_90:10_ – R_+/-_ = 0.9, neutral), 75:25 (AHA-CHT_75:25_ – R_+/-_ = 2.8, positive), and 50:50 (AHA-CHT_50:50_ – R_+/-_ = 8.3, very positive). The charge ratios (R_+/-_) were estimated using the mass ratios AHA-CHT of MPs according to modified model by Rädler *et al.* [32]. Chemical characterization of AHA-CHT was performed by FTIR as described in Section 2.2.1. 

#### 2.2.4. Preparation and Characterization of Microparticles of AHA-CHT (_MP_AHA-CHT)

_MP_AHA-CHT in different mass ratios were obtained by high-speed shearing in an Ultra-Turrax T25 homogenizer (IKA Labortechnik, Staufen, Germany) at 18,000 rpm for 20 min of plane hydrogel swelling in ultrapure water. _MP_AHA-CHT were characterized as described in Section 2.2.2.

#### 2.2.5. Preparation and Characterization of Activated P-PRP 

Plasma rich in platelets and poor in leukocytes (P-PRP) was prepared according to Perez *et al.* [33]. 

P-PRP was prepared from the whole blood (WB) of donors, who were healthy individuals aged between 30 and 40 years old and previously assessed through clinical examination. Human adipose tissue-derived mesenchymal stem cells (h-AdMSCs) were provided by the Umbilical Cord Blood Bank of Haematology and Hemotherapy Center of the University of Campinas. This research was approved by the Ethics Committee of the Medical Sciences School of the University of Campinas (UNICAMP, CAAE: 0972.0.146.000-11).

Briefly, whole blood (WB) was collected into 3.5 mL vacuum tubes (Vacuette®, Greiner Bio-One, Americana, SP, Brazil) containing sodium citrate 3.2% (g/100 mL) as an anticoagulant. WB was firstly centrifuged in a Rotina 380R centrifuge (Hettich^®^ Zentrifugen, Tuttlingen, Germany) at 100× *g* for 10 min at 25°C and the upper layer was collected to obtain P-PRP [33]. The concentrations of platelets in WB (215.625 ± 7.028 pq/mm^3^) and in P-PRP were determined using the ABX Micros ES 60 hematology analyzer (HORIBA ABX Diagnostics, Montpellier, France). P-PRP was obtained with approximately twice the platelet concentration (495.000 ± 18.810 pq/mm^3^).

The activation of P-PRP was done with serum-containing thrombin and CaCl_2_ solution (10% g/100 mL) as agonists applying the following conditions: agonist/P-PRP 20% (mL/100 mL), Serum/CaCl_2_ volumetric ratio 9. These proportions provide fibrin network architecture with thin fibers (approximately 160 nm average radius), which favors the proliferation of h-AdMSCs due to its paracrine nature. Serum was prepared by collecting 5 mL of WB in tubes without anticoagulant. After waiting 30 min to allow clot formation, WB was centrifuged at 2000× *g* for 10 min. The preparation and activation protocols followed Perez et al. [33,34]. 

#### 2.2.6. Association between _A_P-PRP and _MP_AHA

MPs were sterilized by steam in the autoclave at 126 °C (1.5 kgf/cm^2^) for 5 min. Then, the association between _A_P-PRP and _MP_AHA (and _MP_AHA-CHT) was performed in laminar flow cabinet by adding freshly activated P-PRP to MPs, in a volume/mass ratio _A_P-PRP:MP of 200 µL:200 mg. The components were mixed with the tip of a pipette for a short time in 48-well microplates and kept for 45 min for gel structuring. DMEM-LG (Gibco, Grand Island, NY, USA) was then added to the wells for cell culture. 

_A_P-PRP/_MP_AHA and _A_P-PRP/_MP_AHA-CHT were characterized by the release of growth factors, culture of seeded h-AdMSCs and images of the structured gel.

*Release of growth factors.* The release of platelet-derived growth factor AB (PDGF-AB) and transforming growth factor β1 (TGF-β1) was performed after 45 min of gelation of _A_P-PRP alone, _A_P-PRP/_MP_AHA and _A_P-PRP/_MP_AHA-CHT in the presence of the Dulbecco′s Modified Eagle′s Medium with a low glucose concentration (DMEM-LG, Gibco, Grand Island, NY, USA) in 48-well microplates. The microplates were kept in an incubator with 5% CO_2_ over the assays. After sampling, the volume was replaced with fresh medium at 3, 6, 12, 48, and 72 h without removing the gels from the wells. The samples were stored at −80°C until all have been collected. The concentrations of the released GFs were measured using enzyme-linked immunosorbent assay (ELISA) kits (R&D Systems, Minneapolis, MN, USA), according to the manufacturer′s instructions and specifications.

*Culture of seeded h-AdMSCs.* The pre-cultured h-AdMSCs were trypsinized and resuspended in P-PRP to a final cell concentration of 1 × 10^4^ cells/mL. P-PRP containing h-AdMSCs was activated and immediately added to _MP_AHA or _MP_AHA-CHT in 48-well microplates. The association with seeded h-AdMSCs was kept at room temperature for 45 min for consolidation of the structure. After that, DMEM was added. The cultivation of h-AdMSCs was carried out in 48-well tissue culture plates by adding 1 mL of the culture medium DMEM-LG to the wells. The assays were done in quadruplicate. The culture was maintained at 37 °C over 10 days. Cultivations in _A_P-PRP alone were used as control. Cell proliferation was quantified by MTT assay. At 2, 4, 7, and 10 days of cultivation, the samples were removed and transferred to 24-well plates. MTT (1 mL of 1 mg/mL) was then added, and the cultivation proceeded at 37 °C for 4 h. The MTT solution was then discarded, and 1 mL of dimethylsulfoxide (DMSO) was added to dissolve the purple formazan crystals. The samples were shaken at 120 rpm for 30 min to ensure the homogeneous dissolution of the formazan dye, and 200 µL of each sample was then transferred to a 96-well plate. The optical density was measured at 595 nm using a microplate reader (FilterMax F5 Molecular Devices, Sunnyvale CA, USA). The cell number in each well was quantified by a calibration curve of absorbance measured at 595 nm and the cell concentration previously constructed. 

*Images of the structured gel.* Images of the gel structures were obtained by scanning electron microscopy (SEM) 4 days after h-AdMSC cultivation. The samples were fixed in a solution of paraformaldehyde/glutaraldehyde for 2 h and then dehydrated in an ethanol series for 15-min intervals. The samples were dried in a critical point dryer BAL-TEC CPD 030 (BAL-TEC^®^, Schalksmühle, Germany). After gold coating in a Sputter Coater POLARON, SC7620 (VG Microtech, Ringmer, UK), the samples were visualized in a scanning electron microscope Leo440i (LEO Electron Microscopy/Oxford, Cambridge, UK) with an accelerating voltage of 20 kV.

#### 2.2.7. Statistical Analysis

Each experiment was carried out in triplicate unless otherwise specified. All results are presented as the mean ± standard deviation (SD). The experimental data from all the studies were analyzed using analysis of variance (ANOVA). Statistical significance was set to *p*-value ≤ 0.05.

## 3. Results and Discussion

### 3.1. Structural Modifications of HA 

FTIR spectroscopy was used to confirm the formation of AHA and AHA-CHT. The spectrum of AHA showed the characteristic bands of polysaccharides (Supplementary Figure S4) between 3600 and 2800 cm^−1^ attributed to O–H stretching vibration and between 3000 and 2800 cm^−1^ attributed to the C–H stretching vibrations and bands of N–H stretching vibrations overlapped with the O–H bands, as expected. 

The crosslinking was characterized by a decrease in the peaks at the region of 1800–1700 cm^−1^, which is attributed to the stretching vibration of C=O of carboxylic acyl groups (~1700 cm^−1^) and ester groups (1780–1760 cm^−1^). 

The attributions of the absorption bands of AHA-CHT were performed according to Coimbra et al. [35]. The AHA-CHT spectrum showed a broad band between 1700 and 1500 cm^−1^ which was attributed to overlapping of the carbonyl and amide vibrations. The peak at 1598 cm^−1^ assigned to the anti-symmetrical stretching vibration of the carbonyl group of the carboxylate in the AHA appears slightly shifted to the left in the AHA-CHT, indicating the ionic interactions between the carboxylate group of AHA and the NH_3_^+^ group of CHT. Moreover, in the AHA-CHT spectrum a slight displacement of peaks was also observed, attributed to the symmetric stretch vibration of the carboxylate group νs (COO^−^) of AHA from 1407 to 1413 cm^−1^. 

Due to the overlapping, it is difficult to distinguish any sign between the asymmetric and symmetric bending vibrations of the NH_3_^+^ between 1625–1560 and 1550–1505 cm^−1^, respectively [36].

The AHA-CHT spectrum also showed a ratio change in the peaks at 1037 and 1077 cm^−1^, and in the peaks, 1374 cm^−1^ compared to CHT spectra. These changes also suggested the formation of AHA-CHT.

### 3.2. Physicochemical and Mechanical Properties of MPs (_MP_AHA and _MP_AHA-CHT)

The swelling ratio (SR), effective crosslink density (V_e_), molar mass between crosslinks (M_c_) and average particle diameter were determined for MPs (_MP_AHA and _MP_AHA-CHT) and results are shown in Table 1. The values of the swelling ratio (SR) in PBS at 37 °C (Table 1) indicate that MPs were extremely hydrophilic, with the capacity to accommodate high amounts of saline solution in their structure, regardless the mass ratio. 

Cells generally show good spreading, proliferation and differentiation on hydrophilic surfaces. Both HA and CHT have an abundant number of hydrophilic groups, such as hydroxyl, amino, and carboxyl groups, which promote water uptake in their structure. The addition of CHT significantly affected (* *p* < 0.05) the swelling properties compared to _MP_AHA due to the decrease of hydrophilic groups that make hydrogen bonds with water, confirming the ionic crosslinking. Moreover, SR of _MP_AHA-CHT_90:10_ was significantly higher than other mass ratios indicating the lowest crosslinking degree, as expected. 

Flory & Rehner equations were used to determine the effective crosslinking density (V_e_) and the average molecular weight between crosslinks (M_c_) which influence mainly the swelling, mechanical, rheological properties and the stability of hydrogels. The addition of CHT increased V_e_, as expected. Moreover, we observed lower M_c_ values, indicating higher entanglement of chains.

Particle sizes of _MP_AHA-CHT were higher than _MP_AHA, indicating that the coverage with chitosan attenuates the negative charge of the microparticles modifying the AHA surface. Nevertheless, MPs kept mean diameters in the range of 190 to 280 μm, which are within the limits for injectable application (<700 μm) [37].

**Figure 1:**
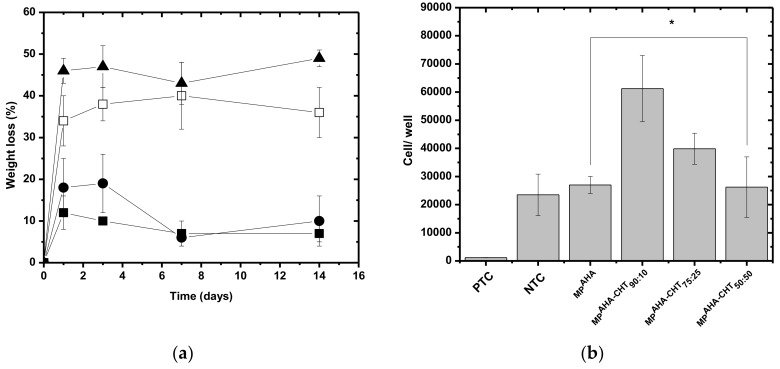
(**a**) The weight loss of the microparticles (MPs) as a function of the time of degradation (T = 37 °C, PBS pH = 7.4). (□) _MP_AHA, (▲) _MP_AHA-CHT_90:10_, (●) _MP_AHA-CHT_75:25_, and (■) _MP_AHA-CHT_50:50_. (**b**) Cytotoxicity expressed as viability of h-AdMSCs exposed to the MPs. Negative toxicity control (NTC) = DMEM with 10% FBS (fetal bovine serum), positive toxicity control (PTC) = DMEM with phenol 0.5%. Mean ± standard deviation n = 3. * The population means were significantly different from the positive control at *p* < 0.05.

The stability results (Figure 1a), expressed as weight loss in PBS at 37 °C, demonstrate that the addition of CHT increased the stability of the MPs. _MP_AHA-CHT_50:50_ shows 14% of weight loss while _MP_AHA shows 34%, confirming the physical crosslinking.

Moreover, except for the _MP_AHA-CHT_90:10_, the other _MP_AHA-CHT were stable (weight loss < 20%) over the 14 days of the assay, showing a capacity to absorb large quantities of PBS without undergoing dissolution.

As seen in Figure 1a, the MPs lost most of their weight on the first day of the assay due to the dissolution of weak bonds between the biopolymer chains. After this first stage, it seems that the remaining biopolymers chains were highly bound together with innumerous ionic interactions and, possibly, other forces like hydrophobic and hydrogen bonds.

The degradation rate was decreased by the addition of CHT, while the _MP_AHA-CHT_90:10_ showed a similar degradation profile of _MP_AHA. 

Figure 1b shows the results of cytotoxicity as assayed by MTT. The MPs were shown not to be toxic against h-AdMSC, compared to positive toxicity controls (PTC = DMEM with phenol 0.5%). Instead, the cells proliferated in the presence of the MPs. The effects of surface charge in MPs were clear. The CHT coverage attenuated the negative charge at the proportions 90:10 and 75:25, but the excess of positive charge in 50:50 also decreased cell proliferation. 

The extrusion force increased with chitosan concentration reaching a maximum at the _MP_AHA-CHT_50:50_ proportion as a consequence of the highest crosslinking degree. 

**Figure 2:**
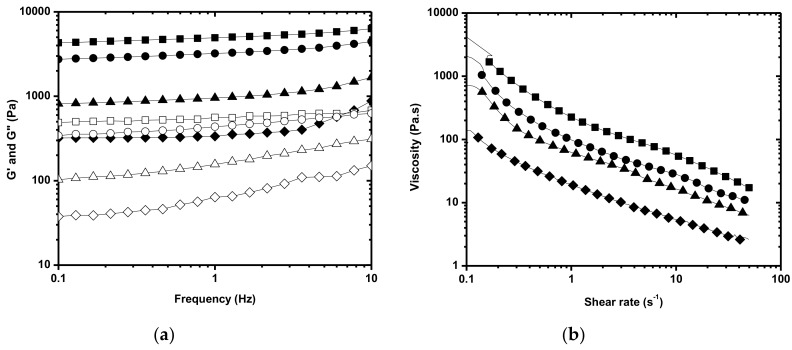
Rheological behavior of _MP_AHA and _MP_AHA-CHT as a function of the mass ratio. (**a**) Oscillation spectrum and (**b**) Flow curve. (♦) _MP_AHA, (▲) _MP_AHA-CHT_90:10_, (●) _MP_AHA-CHT_75:25_, and (■) _MP_AHA-CHT_50:50_. G’ (closed symbol), G” (open symbol).

The rheological behavior of the _MP_AHA and _MP_AHA-CHT determined by oscillatory and steady measurements are shown in Figure 2a,b, respectively. MPs exhibited the gel-like behavior, as analyzed by the storage (G’) and loss moduli (G”), where G’ is higher than G” in all studied frequency ranges and the curves are parallel to the frequency axis. The addition of CHT increased the complex (G*), elastic (G’), and viscous modulus (G”), revealing the crosslinking. The G* values indicate _MP_AHA becomes more resistant to deformation and has a lower fluidity evidenced by tan δ values (damping) with the addition of CHT. Moreover, _MP_AHA and _MP_AHA-CHT exhibited behavior typical of so-called weak gels with tan δ (G”/G’) > 0.1 (Table 1), regardless of their mass ratios.

Regardless of the mass ratio, _MP_AHA-CHT showed G’ values larger than 700 Pa in 1 Hz, which was outside the adequate range (100–700 Pa) for injectable applications. Therefore, _MP_AHA-CHT should be dispersed in the fluid phase or have the crosslinking degree decreased to fit the required range. The flow curve (Figure 2b) showed a shear-thinning behavior for _MP_AHA and _MP_AHA-CHT, which was characteristic of pseudoplastic fluids regardless the mass ratio. Although the addition of CHT increases the viscosity of _MP_AHA-CHT, as expected, in relation to _MP_AHA, the flow behavior index ƞ (ƞ = K·γ^n−1^) was not significantly affected (Table 1).

### 3.3. Rheological and Biological Properties of Associations _A_P-PRP/_MP_AHA and _A_P-PRP/_MP_AHA-CHT

Based on the mechanical properties of MPs (Table 1), the _A_P-PRP/_MP_AHA and _A_P-PRP/_MP_AHA-CHT associations prepared in this work were formed in 200 µL of P-PRP and 200 mg of _MP_AHA or _MP_AHA-CHT. The associations were characterized by rheological properties, release kinetic of PDGF-AB and TGF-β1 and h-AdMSC proliferation. Table 2 summarizes the determined rheological properties of _A_P-PRP/_MP_AHA and _A_P-PRP/_MP_AHA-CHT. 

The interactions between _A_P-PRP and _MP_AHA and _MP_AHA-CHT were evidenced by the values of the complex viscosity (G*), which were higher for the associations than those of _A_P-PRP even with the dilution effect. The G* value indicated _A_P-PRP/_MP_AHA-CHT_50:50_ (G* 185.5 Pa) renders the association more resistant to deformation due to the strong electrostatic interaction (physical crosslinking). The values of tan δ of the associations prepared from AHA-CHT were lower than _A_P-PRP/_MP_AHA and _A_P-PRP, but they were similar to each other.

**Figure 3:**
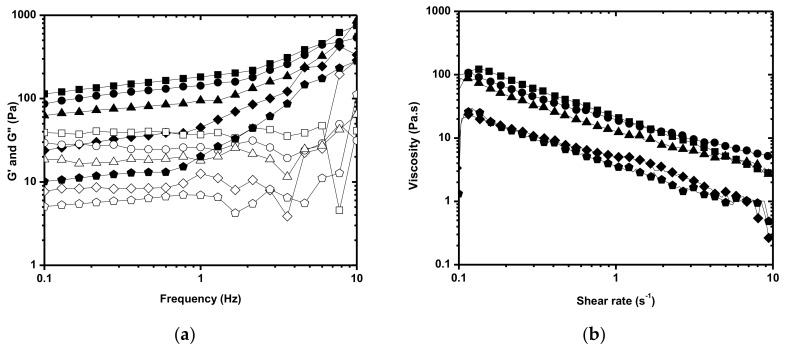
Rheological behavior of the _A_P-PRP/_MP_AHA and _A_P-PRP/_MP_AHA-CHT (200 µL of _A_P-PRP:200 mg of _MP_AHA or _MP_AHA-CHT). (**a**) Oscillation spectrum and (**b**) Flow curve. (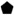) _A_P-PRP, (♦) _A_P-PRP/_MP_AHA, (▲) _A_P-PRP/_MP_AHA-CHT_90:10_, (●) _A_P-PRP/_MP_AHA-CHT_75:25_, and (■) _A_P-PRP/_MP_AHA-CHT_50:50_. G’ (closed symbol), G” (open symbol).

The association with _A_P-PRP as the fluid phase in a 1:1 volumetric ratio _A_P-PRP/_MP_AHA-CHT and _MP_AHA-CHT maintained the weak gel-type behavior (Figure 3a) and the pseudoplastic gel-type behavior (Figure 3b) of the MPs and adequate rheological properties with a reduction in G’ in 1 Hz ≈ 100 Pa as needed, fitting the requirements for injectable use. This volume/ weight ratio _A_P-PRP/_MP_AHA and _A_P-PRP/_MP_AHA-CHT was kept for the biological assays.

**Figure 4:**
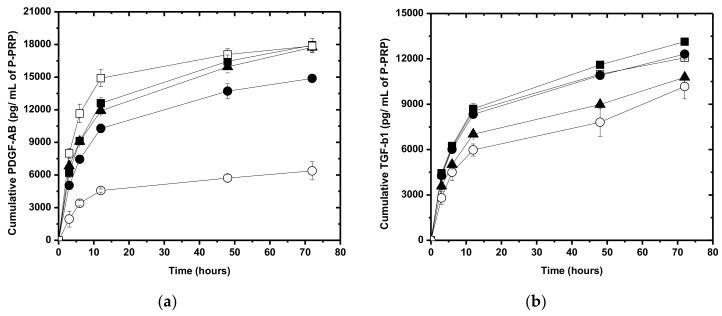
Growth factor release profile from _A_P-PRP/_MP_AHA and _A_P-PRP/_MP_AHA-CHT. (**a**) PDGF-AB and (**b**) TGF-β1. (○) _MP_AHA, (▲) _MP_AHA-CHT_90:10_, (●) _MP_AHA-CHT_75:25_, and (■) _MP_AHA-CHT_50:50_. (□) _A_P-PRP activated with Ca^+2^/ serum was used as control. The concentration of platelets in _A_P-PRP was 495,000 ± 18,810 pq/mm^3^.

The release kinetic of PDGF-AB and TGF-β1 from the _A_P-PRP, _A_P-PRP/_MP_AHA, and _A_P-PRP/_MP_AHA-CHT in DMEM are shown in Figure 4a,b, respectively. The fibrin network from _A_P-PRP was used as a control. In both cases, the profiles are characterized by a predominant diffusion mechanism.

Most of the GFs (~ 70%) from _A_P-PRP occurred after approximately 12 h, related to the maximum release, which is in agreement with the degradation time of the scaffolds observed in Figure 1a. These values are in agreement with in vitro results reported by Tsay et al. [38], showing a burst release of 80% of PDGF-AB and 82% of TGF-β1 after 24 h and a controlled release over the following 14 days. 

The release of PDGF-AB from the _A_P-PRP/_MP_AHA and _A_P-PRP/_MP_AHA-CHT structures were approximately 70% and from the _A_P-PRP was 83%, while the release of TGF-β1 from the _A_P-PRP, _A_P-PRP/_MP_AHA and _A_P-PRP/_MP_AHA-CHT structures ranged from 58 to 70% in 12 h. Maximum TGF-β1 release was not observed until 72 h were reached. 

All _A_P-PRP/_MP_AHA-CHT slowed down the release of PDGF-AB compared to _A_P-PRP alone (Figure 4a), whereas only _A_P-PRP/_MP_AHA-CHT_90:10_ showed the modified release of TGF-β1 (Figure 4b). 

Considering the dilution effects, only _A_P-PRP/_MP_AHA showed lower concentration profiles, releasing a smaller amount of growth factors compared to _A_P-PRP. 

These differences in the release profiles were justified by different chemical interactions of the growth factors with the matrix from the association and/or by its degradation. Due to growth factors are generally alkaline proteins (isoelectric point > 7) at physiological pH, they will have a positive charge that ionically interacts with acidic species [39]. Thus, the elevated density of negative charges of _A_P-PRP/ _MP_AHA increased the magnitude of electrostatic interactions with the growth factors, resulting in a more controlled release [40].

In contrast, the release from _A_P-PRP/ _MP_AHA-CHT was governed by the degradation rate because the ionic crosslinking reduced the number of exposed negative charges, thereby compromising the electrostatic interactions. 

To evaluate cell proliferation, the purified population of h-AdMSC was firstly characterized by immunophenotyping cell surface markers such as CD29^+^, CD73^+^, CD90^+^, CD105^+^, and HLA-ABC^+^ or the absence of HLA-DR^+^, and CD34^-^, CD45^-^ by flow cytometry (data not shown).

MTT assay quantified the proliferation of the h-AdMSCs in the associations _A_P-PRP/_MP_AHA and _A_P-PRP/_MP_AHA-CHT. The cell proliferation, measured by absorbance at 540 nm, was proportional to the cell concentration. _A_P-PRP was used as control.

**Figure 5:**
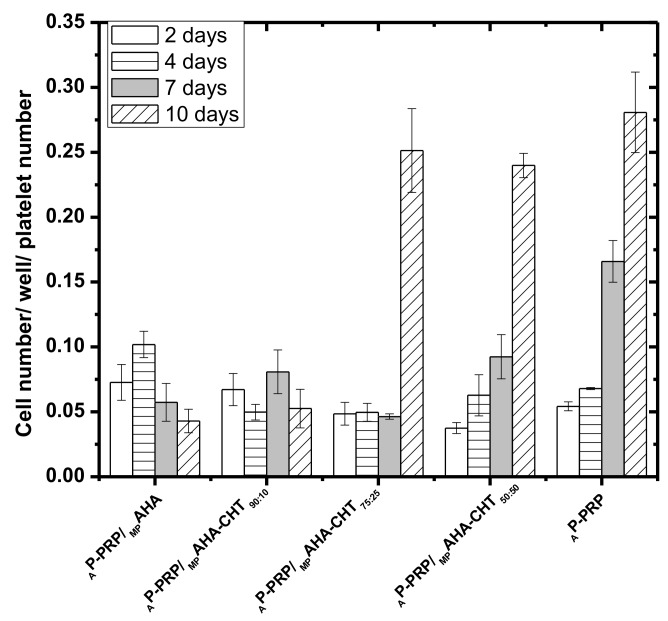
The proliferation profiles of h-AdMSCs cultured in the associations and _A_P-PRP as a function of time. _A_P-PRP was used as control. The concentration of platelets in _A_P-PRP was 495.000 ± 18.810 pq/mm^3^.

**Figure 6:**
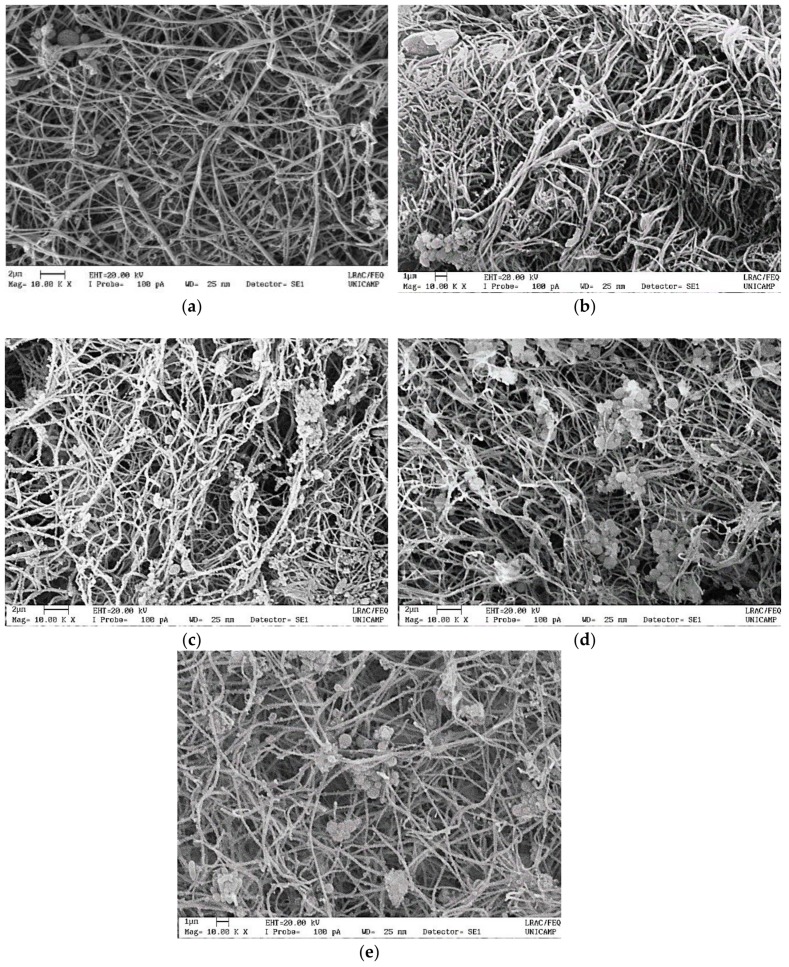
Scanning electron microscopic micrographs of (**a**) _A_P-PRP, (**b**) _A_P-PRP/ _A_P-PRP/_MP_AHA, (**c**) _A_P-PRP/_MP_AHA-CHT_90:10_, (**d**) _A_P-PRP/_MP_AHA-CHT_75:25_, and (**e**) _A_P-PRP/_MP_AHA-CHT_50:50_ after four days of cultivation of h-AdMSCs.

Figure 5 shows the results of h-AdMSC cultivation on _A_P-PRP/MP and _A_P-PRP, and the micrographs of Figure 6 show the structures after 4 days of cultivation in DMEM at 37 °C. 

The cell number per well per platelet number determined after two days exceeded the number of seeded cells (1.0 × 10^4^ cells/well/platelet number) in all associations, meaning that the cells maintained in the matrices retained their viability. The results also demonstrate that the associations provided favorable microenvironments to support the cultivation and proliferation of h-AdMSC in vitro cultures because in all associations the cell viabilities were remarkable up to 10 days.

Except in _A_P-PRP/_MP_AHA and _A_P-PRP/_MP_AHA-CHT_90:10_, the proliferations were statistically (*p < 0.05) similar to _A_P-PRP after 10 days of cultivation. 

Although for _A_P-PRP/_MP_AHA, _A_P-PRP/_MP_AHA-CHT_90:10_, and _A_P-PRP/_MP_AHA-CHT_75:25_ the cell number determined after two days (3.5 × 10^4^ cells/ well/platelet number) exceeded the number of seeded cells (~ 1 × 10^4^ cells/well/platelet number), the data show a long adaptation phase, or lag time with incipient proliferation up to seven days, which was attributed to the negative charge of the associations. On the 10^th^ day, we observed an abrupt cell growth for _A_P-PRP/_MP_AHA-CHT_75:25_ with the cell number statistically (*p < 0.05) similar to _A_P-PRP.

_A_P-PRP and _A_P-PRP/_MP_AHA-CHT_50:50_ showed an exponential growth after two days confirming the influence of the negative charge attenuation on cell cultivation. These results suggest that CHT coverage favored h-AdMSC proliferation and the negative charge attenuation reduced the lag-phase time.

Figure 6b–e shows that the gel matrix structure provided by the associations was similar to that of the fibrin network (_A_P-PRP) Figure 6a. The fibers were approximately 160 nm, and were located preferably on the surface of the MPs, providing a large area covered by cell adhesion and proliferation. The porous structure of the MPs could not be observed because they collapsed under dehydration, which was required to obtain the images. It also was observed that the cells were homogeneously spread on the fibrin fibers in the associations and in _A_P-PRP. 

## 4. Conclusions

In this work, we studied a matrix consisted of platelets, fibrin network (natural scaffold) and HA crosslinked microparticles. We considered the stability, the rheological properties, the toxicity, the superficial area and the superficial charge of the matrix as the critical properties for the ‘association’ to be successful. These properties were evaluated and the results showed that the matrix is promising for orthopedic tissue engineering. The auto-crosslinked microparticles approach represents a strategy for controlling the hyaluronic acid structure for association with P-PRP (pure PRP, i.e., plasma enriched with platelets and poor in leukocytes). This approach is safe, allows easier protocol standardization and may be useful for clinical applications. Moreover, the microparticles improved the physicochemical and mechanical properties of activated P-PRP and provided a large surface area for cell adhesion and proliferation. The association formed matrix (microparticles + fibrin network + platelets) with the fibrin network preferentially on the surface of the microparticles. The coverage with chitosan attenuated the negative charge of the microparticles, producing proliferation profiles similar to the activated P-PRP alone (200 μL of _A_P-PRP) even with the dilution effects of 1:1 in the associations (200 μL of _A_P-PRP:200 μg) of MPs. Therefore, the reduction of negative charge density of the microparticles favored cell proliferation. The possibility to obtain injectable formulations opens perspectives on the use of these biomaterials in non-surgical applications of regenerative medicine, especially in orthopedics.

## Figures and Tables

**Table 1:** Physicochemical and mechanical properties of the microparticles.

**Physicochemical Properties**						
**Microparticles**	**Swelling Ratio** SR=Ws/Wd	**V_e_**	**M_c_**		**Particle Size (µm)**	**Weight Loss in 24 h (%)**
_MP_AHA	61 ± 8	1.28 × 10^−6^	1,015,979		190 ± 6	34 ± 6
_MP_AHA-CHT_90:10_	47 ± 1	2.79 × 10^−5^	155,067		273 ± 4	46 ± 3
_MP_AHA-CHT_75:25_	38 ± 2	3.72 × 10^−5^	116,635		280 ± 4	18 ± 7
_MP_AHA-CHT_50:50_	35 ± 1	4.18 × 10^−5^	103,614		263 ± 4	14 ± 3
**Mechanical Properties**						
**Microparticles**	**G’ (Pa)**	**G” (Pa)**	G* (Pa)	**tan δ (=G”/G’) in 1 Hz**	**n** **ƞ = K·γ^n−1^**	**Extrusion Force (N)**
_MP_AHA	335.8	63.8	341.8	0.19	0.39	20 ± 1
_MP_AHA-CHT_90:10_	954.1	156.4	966.8	0.16	0.24	14 ± 2
_MP_AHA-CHT_75:25_	3211	436.4	3240.5	0.14	0.27	19.9 ± 0.6
_MP_AHA-CHT_50:50_	4933	560.2	4964.7	0.11	0.32	26.7 ± 0.4

**Table 2:** Mechanical properties of the associations _A_P-PRP/_MP_AHA and _A_P-PRP/_MP_AHA-CHT.

Microparticles	G’ (Pa)	G” (Pa)	G* (Pa)	tan δ (=G”/G’) in 1 Hz	n ƞ = K·γ^n−1^
_A_P-PRP	20.17	6.94	21.3	0.34	0.1953
_A_P-PRP/_MP_AHA	45.03	12.54	46.7	0.28	0.2456
_A_P-PRP/_MP_AHA-CHT_90:10_	94.66	18.03	96.4	0.19	0.3381
_A_P-PRP/_MP_AHA-CHT_75:25_	142.8	26.1	145.2	0.18	0.2412
_A_P-PRP/_MP_AHA-CHT_50:50_	181.8	36.86	185.5	0.20	0.1173

## Supplementary Materials

Supplementary materials can be found at https://www.mdpi.com/2073-4360/11/10/1568/s1.

## Abbreviations

PRP = platelet-rich plasma; HA = hyaluronic acid; P-PRP = pure PRP (plasma enriched with platelets and poor in leukocytes); _A_P-PRP = activated pure platelet rich plasma; AHA = auto-crosslinked hyaluronic acid; _MP_AHA = microparticles of auto-crosslinked hyaluronic acid; CHT = chitosan; MPs = microparticles; _A_P-PRP/_MP_AHA = activated pure platelet rich plasma associated to microparticles of auto-crosslinked hyaluronic acid; _A_P-PRP/_MP_AHA-CHT = activated pure platelet rich plasma associated to microparticles of auto-crosslinked hyaluronic acid and chitosan; PDGF-AB = platelet-derived growth factor AB; TGF-β1 = transforming growth factor β1; h-AdMSCs = human adipose-derived mesenchymal stem cells.
