# Supplementary material for: Association of Platelet-Rich Plasma and Auto-Crosslinked Hyaluronic Acid Microparticles: Approach for Orthopedic Application
Source: Polymers (Basel). 2019 Sep 26;11(10):1568. doi: 10.3390/polym11101568 (PMC6835642; doi:10.3390/polym11101568)
Supplement: Supplementary file 1 [file polymers-11-01568-s001.pdf]

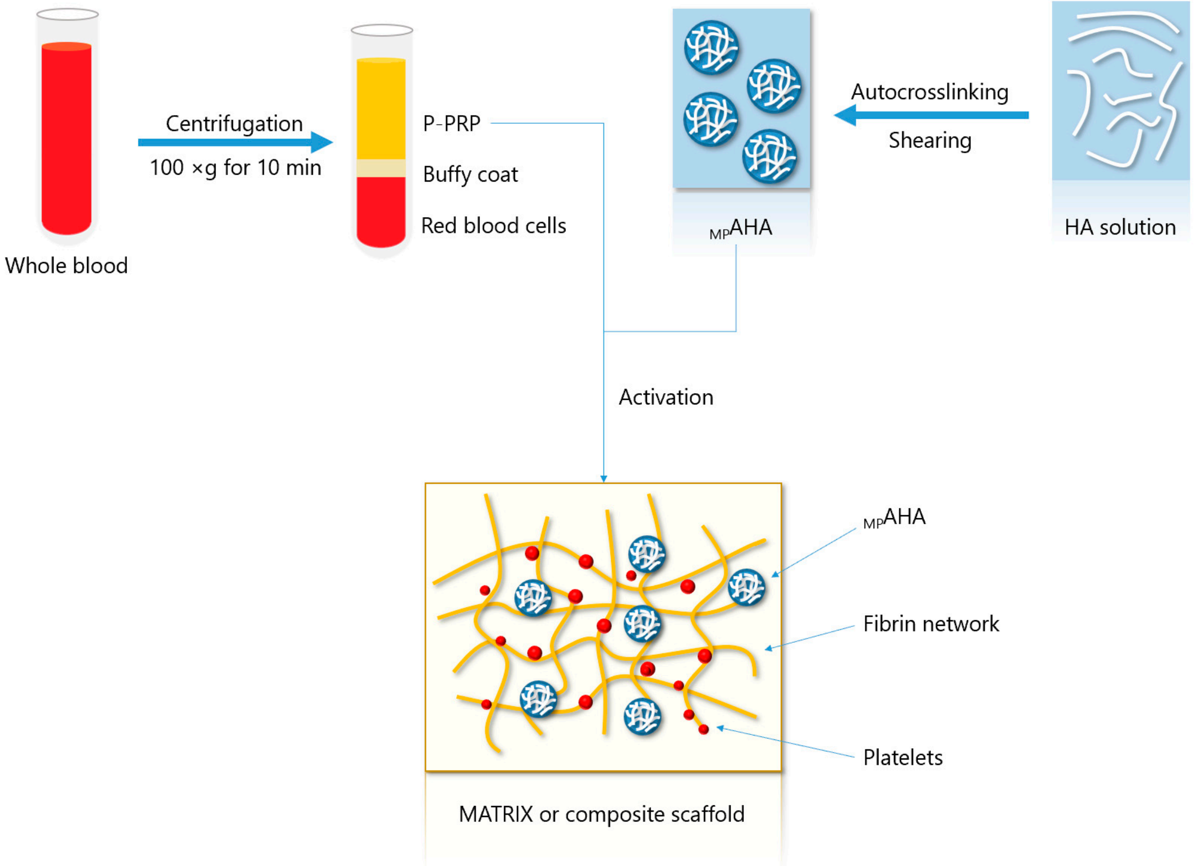

Figure S1. Diagram representing the matrix formation.

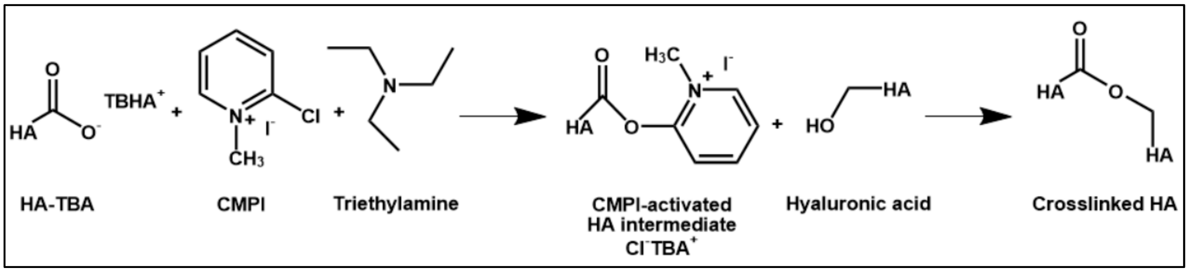

Figure S2. Autocrosslinking reaction using 2-chloro-1-methyl pyridinium iodide (CMPI)-activated hyaluronic acid (HA) intermediate. TBA^+ = tetrabutylammonium ion.

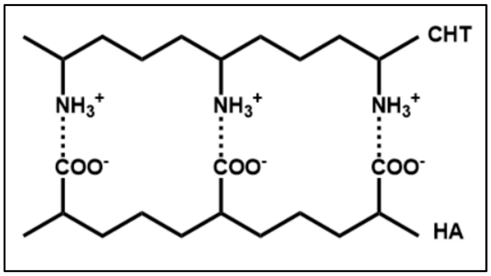

Figure S3. Complex coacervation between chitosan (CHT) and hyaluronic acid (HA).

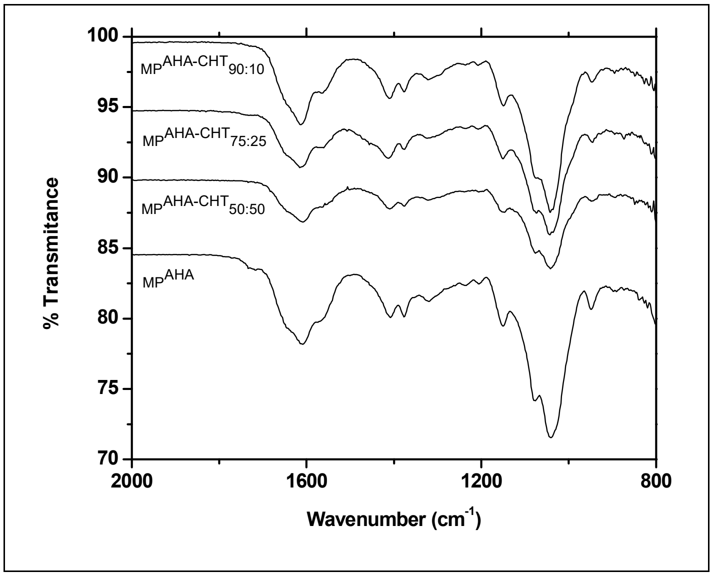

Figure S4. FTIR-ATR spectra of microparticles of autocrosslinked hyaluronic acid ($_{MP}$AHA) and microparticles of autocrosslinked hyaluronic acid – chitosan ($_{MP}$AHA-CHT).
